# Alterations of gut microbiota in biopsy-proven diabetic nephropathy and a long history of diabetes without kidney damage

**DOI:** 10.1038/s41598-023-39444-4

**Published:** 2023-07-27

**Authors:** Xiao Lu, Junjun Ma, Rongshan Li

**Affiliations:** 1 Department of Nephrology, Fifth Hospital of Shanxi Medical University (Shanxi Provincial People’s Hospital), Taiyuan, China; 2 Department of Thoracic Surgery, Fifth Hospital of Shanxi Medical University (Shanxi Provincial People’s Hospital), Taiyuan, China

**Keywords:** Biomarkers, Nephrology

## Abstract

The gut microbiota is closely related to parenteral noncommunicable diseases through intestinal immunity and plays an important role in the occurrence of diabetes and diabetic nephropathy. The aim of the study was to understand the gut–kidney axis by an analysis of gut microbiota composition among patients with biopsy-proven diabetic nephropathy (DN), patients with type 2 diabetes for more than 10 years without kidney damage (DM), and healthy controls (NC). Thirty-five DN patients, 40 DM patients and 40 healthy subjects matched by age and sex were enrolled between January 2022 and December 2022. Baseline information and clinical parameters were collected. 16S rDNA sequencing was performed to characterize the gut microbiome and identify gut microbes that were differentially abundant between patients and healthy controls. The relationship between the relative abundance of specific bacterial taxa in the gut and clinical phenotype and pathological indicators was evaluated. Substantial differences were found in the richness of the gut microbiota and the variation in the bacterial population among DN patients, DM patients and healthy controls. DM patients could be accurately distinguished from age- and sex-matched healthy controls by variations in g_Clostridium-XVIII (AUC = 0.929), and DN patients could be accurately distinguished from age- and sex-matched healthy controls by variations in g_Gemmiger (AUC = 0.842). DN patients could be accurately distinguished from age- and sex-matched DM patients by variations in g_Flavonifractor or g_Eisenbergiella (AUC = 0.909 and 0.886, respectively). The gut microbiota was also closely related to clinical phenotypes and pathological indicators. The study of gut microbiota composition was explored to determine its relationship to the occurrence of DN and a long history of diabetes without kidney damage. The renal pathological progression of DN may be delayed by regulating changes in the gut microbiota.

## Introduction

With the increasing prevalence of diabetes, the number of patients with diabetic nephropathy (DN) has also increased significantly. The results of an epidemiological survey in China from 2015 to 2017 showed that the prevalence of diabetes in adults was 11.2%^1^. As a serious microvascular complication of diabetes, DN has become the main cause of end-stage renal disease (ESRD) worldwide^2–4^. There are about 500–1000 species of bacteria in the human gastrointestinal tract, with a number of 10^14^ CFU, which are vividly referred to as acquired "organs"^5^. They assist the host in maintaining normal physiological functions, including absorbing energy, producing vitamins and basic active molecules, biological antagonism, immune regulation. Its composition and number have maintained a dynamic balance with the host in evolution^6^. A large number of studies have shown that gut microbiota can participate in the occurrence and development of diseases by regulating host energy metabolism, systemic inflammatory response, and the secretion of intestinal hormones. Gut microbiota also play an important role in a variety of kidney diseases, and the endotoxins, proteins and some metabolites produced by them have certain effects on the kidney through the gut-kidney axis^7^. By observing the gut microbiota of patients with diabetic nephropathy confirmed by renal biopsy and patients with long-term diabetes without kidney damage, this study aimed to explore the imbalance pattern and functional changes in the two disease states and establish a classification algorithm model to distinguish the two by gut microbiota. We also explore the correlation between gut microbiota and clinical phenotypes and pathological indexes.

## Methods

### Study participants

The retrospective study included 35 patients with diabetic nephropathy confirmed by renal biopsy, 40 patients with type 2 diabetes for more than 10 years without kidney damage, and 40 healthy subjects matched by age and sex. We enrolled patients and healthy controls in Shanxi Provincial People’s Hospital between January 2022 and December 2022. In the DN group, inclusion criteria were as follows:

- 18–65 y of age;
- Diagnosed with type 2 diabetes;
- Diabetic nephropathy diagnosed based on renal puncture pathological examination;
- No evidence of primary renal disease;
- Estimated glomerular filtration rate(eGFR) ≥ 60 ml/min/1.73 m^2^;
- Informed consent can be signed.

In the T2DM group, inclusion criteria were as follows:

- 18–65 y of age;
- The duration of type 2 diabetes was more than 10 years;
- No diabetic microvascular complications, including diabetic retinopathy and renal damage (eGFR ≥ 60 ml/min/1.73 m^2^ and urine albumin creatinine ratio (UACR) < 30 mg/g);
- Informed consent could be signed.
- The exclusion criteria for both groups included:
- Severe heart, lung, liver, kidney and other organ dysfunction.
- Malignant tumor, autoimmune disease or gastrointestinal disease; Use of antibiotics, preparations of live bacteria, lactulose or immunosuppressants within nearly a month.
- Pregnant or lactating women.

The healthy controls were from the Physical Examination Center of Shanxi Provincial People’s Hospital and were 18–65 y of age. The results indicated that they were healthy, had no gastrointestinal symptoms and had not taken any drug in the past one month.

All study procedures complied with the ethical guidelines of the

Declaration of Helsinki.The studies involving human participants were reviewed and approved by the Biomedical Ethics Committee of Shanxi Provincial People’s Hospital (No. 2022–222). The patients/participants provided their written informed consent to participate in this study.

### Fecal sample collection and DNA extraction

Fresh fecal samples were collected from the selected individuals. The fecal samples were discharged into a sterile stool box and transferred to a − 80 °C refrigerator for storage within 2 h after sampling. DNA was extracted from the samples using the QIAamp PowerFecal DNA Kit. Agarose gel electrophoresis was used to analyze DNA integrity, NanoDrop was used to measure the purity, and DNA concentration was accurately quantified with Qubit.

### 16S rDNA gene-targeted amplification and library construction

High-fidelity DNA polymerase was used to amplify the V3-V4 variable region of DNA by two-step PCR amplification-specific amplification and the addition of tag and joint sequences. An FC Magnetic Beads Kit (Enlighten) was used to purify and recover the product. Qubit4.0 was used to quantify the purified library, Qsep100 was used to check whether the length of the library was as expected, and each sample was diluted to 4 nM. The hybrid library was prepared and denatured by DNA, and at least a 5% Phix library was added to balance the library polymorphism. Sequencing was carried out on an Illumina MiSeq sequencer using the PE300 strategy.

### Processing of sequencing data

The quality of the original data was controlled by QIIME2, low-quality sequences and joint sequences were filtered by Trimmomatic software, and chimeric sequences were removed by USEARCH software. OTU clustering was carried out by UPARSE software, and the bacterial 16S region was compared to the RDP database.

### Statistical analysis

Statistical analysis was performed using R software (version 4.0.2). Amplicon analysis included only microorganisms that existed in ≥ 12% of samples. Alpha diversity was used to measure the richness of microbial species in a single sample. Beta diversity was used to measure the similarity of microbiota composition among different samples. In this study, principal coordinate analysis (PCoA) was used to analyze the β diversity of the microbiota. Through PCoA, we observed whether there was an overall deviation of microbiota between the disease group and the healthy population. LEfSe, or linear discriminant analysis effect size analysis, can find biomarkers with statistically significant differences between groups. This method was used to compare the gut bacteria with statistically significant differences between each disease group and the normal control group. Random forest is an integrated machine learning method that can obtain the final result by establishing multiple decision trees and voting (average) on the prediction results of each decision tree, which has the same function as the decision tree to complete the classification and regression tasks. In this study, the random forest prediction model was established by omicStudio software. The classification efficiency of the model was evaluated by the area under the curve (AUC) of the working characteristic curve (ROC). The prediction performance of the model was analyzed. The discriminant value was low when AUC < 0.70, good when 0.7 ≤ AUC ≤ 0.9, and high when AUC > 0.9. All heatmaps were visualized by the package pheatmap (version 1.0.12). The clinical data of patients with DN and DM and healthy controls were expressed as the mean ± SD or median, and the numerical data were expressed as percentages. A t test or nonparametric test was used to compare continuous variables. Chi-square tests were used to compare categorical variables. The statistical analysis was performed using SPSS version 26.0.

## Results

### General characteristics of all participants

We included 35 patients with DN confirmed by renal biopsy with a median age of 54.09 years and 40 DM patients with a median age of 58.83 years. The baseline characteristics of the DN, DM and healthy control (NC) groups are summarized in Table 1.

**Table 1:** Baseline characteristics of participants.

	DM (n = 40)	DN (n = 35)	NC (n = 40)	*P* value
Age, years	58.83 ± 3.71	54.09 ± 6.39	54.44 ± 7.21	0.114
Gender, n (%)				
Female	13(33)	15(42)	13(33)	0.795
Male	27(67)	20(58)	27(67)	
DM duration, years	15.05 (11.5, 16.83)	16 (15, 19)	0 (0, 0)	< 0.001
Body mass index, kg/m^2^	23.45 ± 2.67	26.12 ± 4.77	23.51 ± 2.07	0.045
Serum creatinine, umol/L	60.74 ± 16.46	90.07 ± 22.51	69.22 ± 10.24	< 0.001
Fasting blood glucose, mmol/L	8.25 (7.18, 9.35)	6.83 (5.38, 9.52)	4.97 (4.86, 5.31)	< 0.001
HbA1C, %	7.8 (7.47, 8.93)	7.8 (7.2, 9.1)	5.3 (5.1, 5.6)	< 0.001
UACR, mg/g	7.03 (4.82, 12.99)	3092 (1619.78, 5161.6)	5.9 (5.4, 6.7)	< 0.001
eGFR, ml/min*1.73m^2^	120.39 ± 25.05	81.77 ± 31.45	106.48 ± 18.04	< 0.001

*DN* diabetic nephropathy, *NC* healthy controls, *HbA1c* glycosylated hemoglobin, *UACR* urine albumin creatinine ratio, *eGFR* estimated glomerular filtration rate.

DM type 2 diabetes mellitus for more than 10 years without kidney damage.

### Pathological classification of diabetic nephropathy

According to the pathological grade of diabetic nephropathy, the severity of pathological changes was evaluated by glomerular volume (G), glomerular basement membrane (GBM), mesangial matrix (M), glomerulosclerosis (S) and interstitial lesions (I). G0 represents no significant increase in glomerular volume, and G1 represents an increase in glomerular volume. GBM1 represents mild thickening of the glomerular basement membrane, and GBM2 represents significant thickening of the glomerular basement membrane. M1 represents mild proliferation of the mesangial matrix, M2 represents significant widening of the mesangial matrix, and M3 represents the formation of one or more tuberous sclerosis (Kimmelstiel-Wilson nodules). S1 represents less than 50% glomerulosclerosis and S2 represents more than 50% glomerulosclerosis. I1 represents mild renal interstitial lesions, I2 represents moderate renal interstitial lesions, and I3 represents severe renal interstitial lesions. Among the 35 diabetic nephropathy patients, there were 5 cases of G0 and 30 cases of G1, 23 cases of GBM1 and 12 cases of GBM2, 4 cases of M1, 4 cases of M2 and 27 cases of M3, 30 cases of S1 and 5 cases of S2, 17 cases of I1, 16 cases of I2 and 2 cases of I3, as shown in Table 2.

**Table 2:** Pathological classification of diabetic nephropathy.

DN	G	GBM	M	S	I
1	1	1	1	1	2
2	1	2	3	1	1
3	1	1	2	1	1
4	1	1	3	1	1
5	1	1	3	1	1
6	1	1	3	1	2
7	1	1	3	1	1
8	1	1	3	1	2
9	1	2	3	1	2
10	0	2	3	2	2
11	1	1	1	1	2
12	1	2	3	1	1
13	1	1	2	1	1
14	1	1	3	1	1
15	1	1	3	1	1
16	1	1	3	1	2
17	1	1	3	1	1
18	1	1	3	1	2
19	1	2	3	1	2
20	0	2	3	2	2
21	0	2	3	2	3
22	1	1	1	1	2
23	1	2	3	1	1
24	1	1	2	1	1
25	1	1	3	1	1
26	1	1	3	1	1
27	1	1	3	1	2
28	1	1	3	1	1
29	1	1	3	1	2
30	1	2	3	1	2
31	0	2	3	2	2
32	0	2	3	2	3
33	1	1	1	1	2
34	1	2	3	1	1
35	1	1	2	1	1

*DN* diabetic nephropathy, *G* glomerular volume, *GBM* glomerular basement membrane, *M* mesangial matrix, *S* glomerulosclerosis, *I* interstitial lesions.

### Differences in fecal microbiota diversity between T2DM and DN subjects

**Figure 1:**
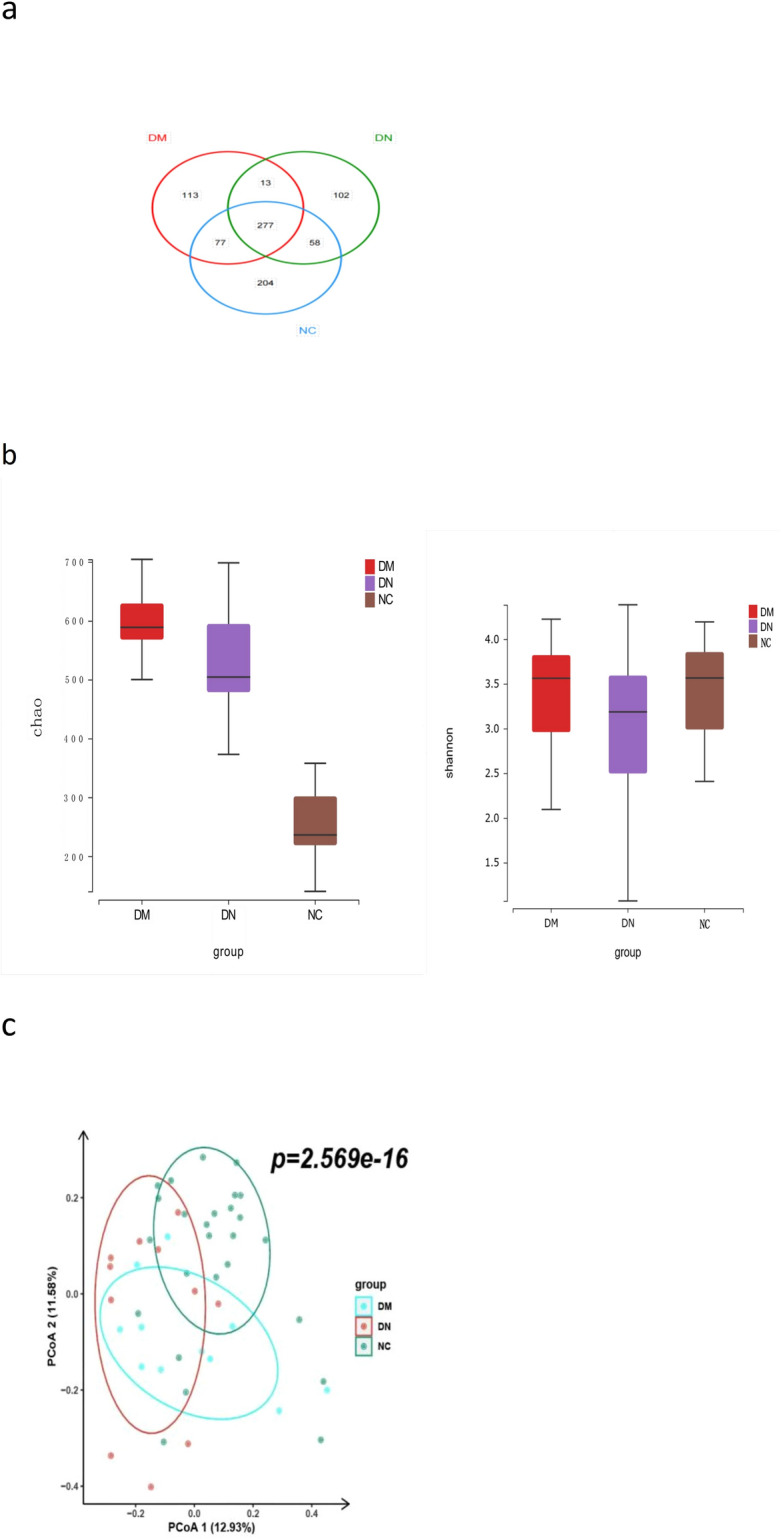
Gut microbiota compositions differed among DN, DM, NC groups. (**a**) Venn diagram of DN, DM, and NC; (**b**) Analysis of Alpha diversity in DN, DM, and NC; (**c**) Beta diversity analysis of DN, DM, and NC. DN diabetic nephropathy, DM type 2 diabetes mellitus for more than 10 years without kidney damage, NC healthy controls, OTUs operational taxonomic units.

A total of 844 OTUs were obtained at a 97% homology cut-off. The NC group showed the largest number of OTUs. As shown in the Venn diagram (Fig. 1a), the number of OTUs in common between the NC and DM groups was 354, while the DM and DN groups had 290 OTUs in common. The DM group had 113 specific OTUs, and the DN group had 102 specific OTUs that the other two groups did not share.

The Chao index of the OTU profile was used to evaluate gut microbiota richness, and the Shannon index was used to estimate community diversity. The differences in community richness were significant in the three groups (DM vs. DN Chao *P* = 0.005, DM vs. NC Chao *P* < 0.001, DN vs. NC Chao *P* < 0.001), but no different diversity of OTUs was found in the three groups (Shannon *P* > 0.05) (Fig. 1b). Principal coordinates analysis (PCoA) based on Bray–Curtis dissimilarity at the OTU level showed that the microbiota compositions of NC, DM, and DN patients were significantly different, as shown in Fig. 1c (P = 2.569e^−16^). The fecal microbiota of patients with DM and DN and the NC subjects was clearly separated along PCoA2 and PCoA1, which explained 11.58% and 12.93% of the total variation, respectively. Together, these data suggest that the fecal microbial structure was significantly altered in patients with DM and DN compared to that in NC subjects in the presence of OTUs.

**Figure 2:**
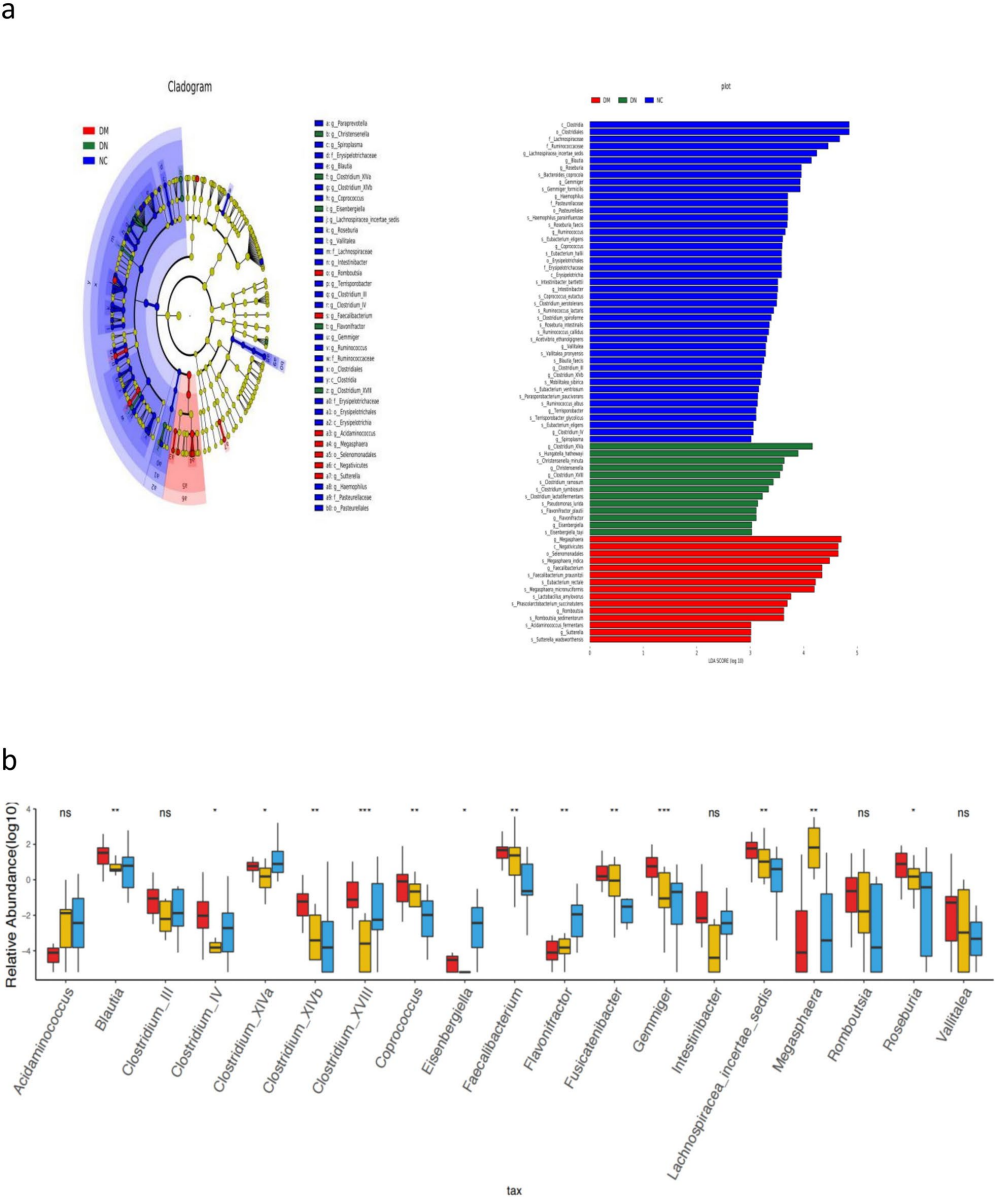
Taxonomic differences in the fecal microbiota exhibited by patients with diabetic nephropathy (DN), type 2 diabetes mellitus for more than 10 years without kidney damage (DM) and healthy control (NC) subjects. (**a**) A Linear discriminant analysis (LDA; (log10) > 3) and effect size (LEfSe) analysis revealed significant differences (*P* < 0.05) in the fecal microbiota exhibited by the DM (reg score),DN(green score) and NC (blue score) groups. (**b**) This analyse revealed the most differentially abundant taxa at the genus level of bacterial between DM (yellow),DN (blue) and NC (red) groups. *P + 0.05 + **P + 0.01.

To determine different taxa from the phylum to genus level among the three groups, the linear discriminate analysis (LDA) effect size (LEfSe) algorithm was used (Fig. 2a). LEfSe showed that compared with the healthy control group, the number of gut microbiota in 2 classes, 3 orders and 5 families was significantly decreased in the DM and DN groups from the phylum to family level, while the number of Negativicutes and Selenomonadales was increased in the T2DM group. At the genus level, the number of Romboutsia, Faecalibacterium, Acidaminococcus, Megasphaera and Sutterella species in the DM group was significantly increased (all *P* < 0.05). The numbers of Christensenella, Clostridium-XIVa, Eisenbergiella, Flavonifractor and Clostridium-XVIII in the DN group were significantly increased (all *P* < 0.05), while the numbers of butyric-producing bacteria, Bacillus, Enterobacter, Trichospira and Rosacella were significantly decreased in the DM group and DN group (P < 0.05). At the genus level, Clostridium XVIII and Gemmiger were significantly decreased in the DM and DN groups (Fig. 2b).

**Figure 3:**
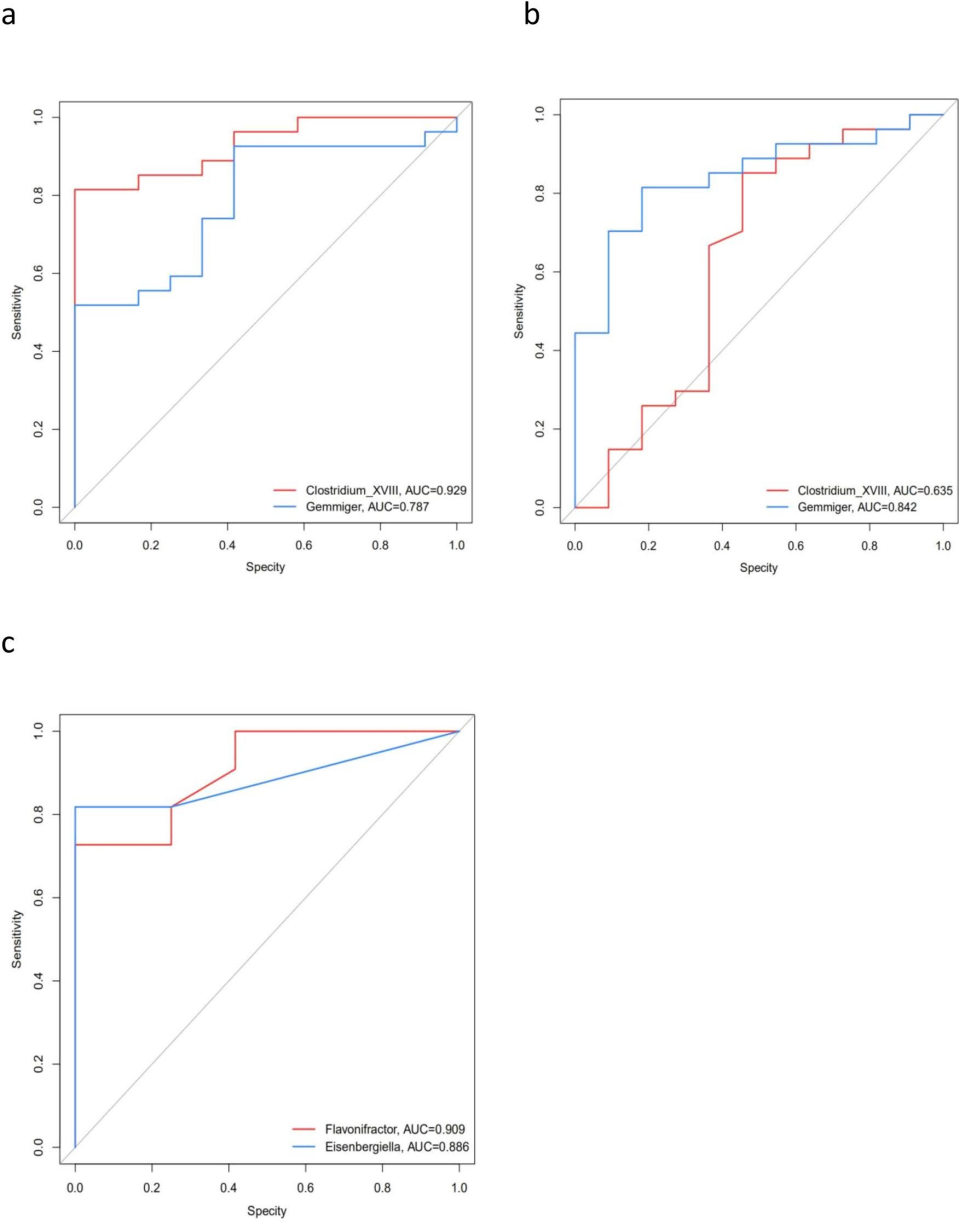
Receiver operating characteristic curve (ROC) analysis of the sensitivity and specificity of the differentially abundant genera as diagnostic factors for type 2 diabetes mellitus for more than 10 years without kidney damage(DM) and diabetic nephropathy(DN).(**a**) ROC curve classifying DM from healthy controls(NC), based on g_Clostridium-XVIII; (**b**) ROC curve classifying DN from NC, based on g_Gemmiger; (**c**) ROC curve classifying DN from DM, based on g_Flavonifractor and g_Eisenbergiella.

To illustrate the microbial signature of DM and DN and further explore the potential of the gut microbiome in DN identification, receiver operating characteristic (ROC) curves for classifying DM from NC, DN from NC and DN from DM were developed. We could detect DM individuals accurately based on only one genus (g_Clostridium-XVIII), as indicated by an area under the receiver operating curve (AUC) of up to 0.929 (Fig. 3a). Similarly, the richness of one genus (g_Gemmiger) was effective in distinguishing DN individuals from healthy controls, showing an AUC of 0.842 (Fig. 3b). The richness of two genera (g_Flavonifractor and g_Eisenbergiella) was effective in classifying DN subjects from DM subjects, showing AUCs of 0.909 and 0.886, respectively (Fig. 3c).

### Potential correlations between the differences in gut microbiota and clinical characteristics

**Figure 4:**
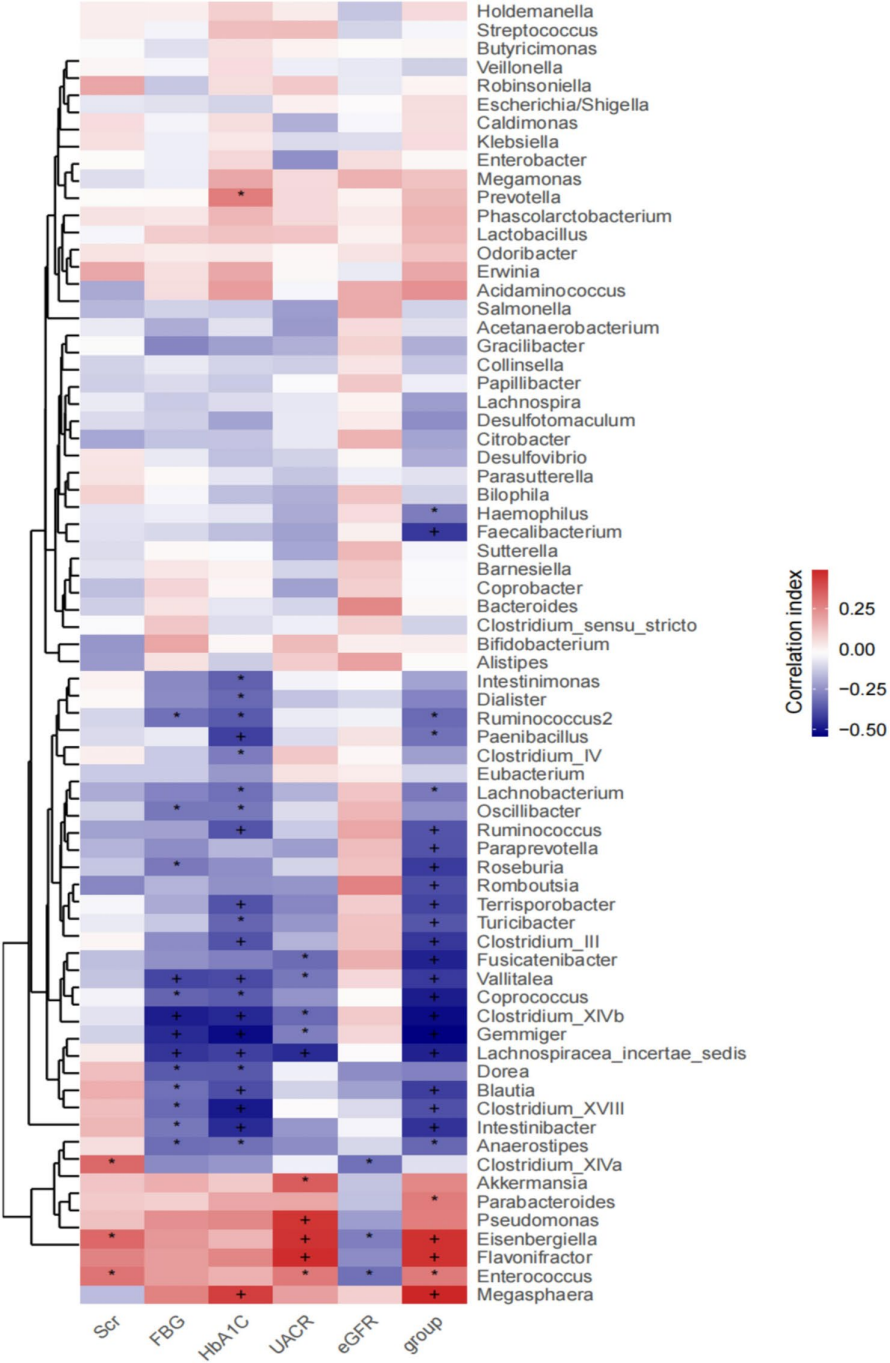
Heatmaps showing correlations between differentially abundant microbiota genera and clinical parameters. SCr—serum creatinine; FBG—fasting blood glucose; HbA1c—glycosylated 16 hemoglobin; UACR—urine albumin creatinine ratioe; GFR—estimated glomerular filtration rate; Spearman test, *P < 0.05, ^+^*P* < 0.01.

Spearman correlation analysis was used to further evaluate the correlation between gut microbiota differences and clinical characteristics. At the genus level, Gemmiger, Clostridium XlVb, Lachnospiracea incertae sedis and Vallitalea showed strongly negative correlations with glycosylated haemoglobin (HbA1c) and fasting blood glucose (FBG) levels, while Megasphaera had a positive correlation with HbA1c levels. Pseudomonas, Eisenbergiella and Flavonifractor showed markedly positive correlations with the urine albumin/creatinine ratio (UACR). Enterococcus, Clostridium XlVa and Eisenbergiella exhibited a negative correlation with the estimated glomerular filtration rate (eGFR) (Fig. 4).

### Correlation between gut microbiota and pathological types of DN

**Figure 5:**
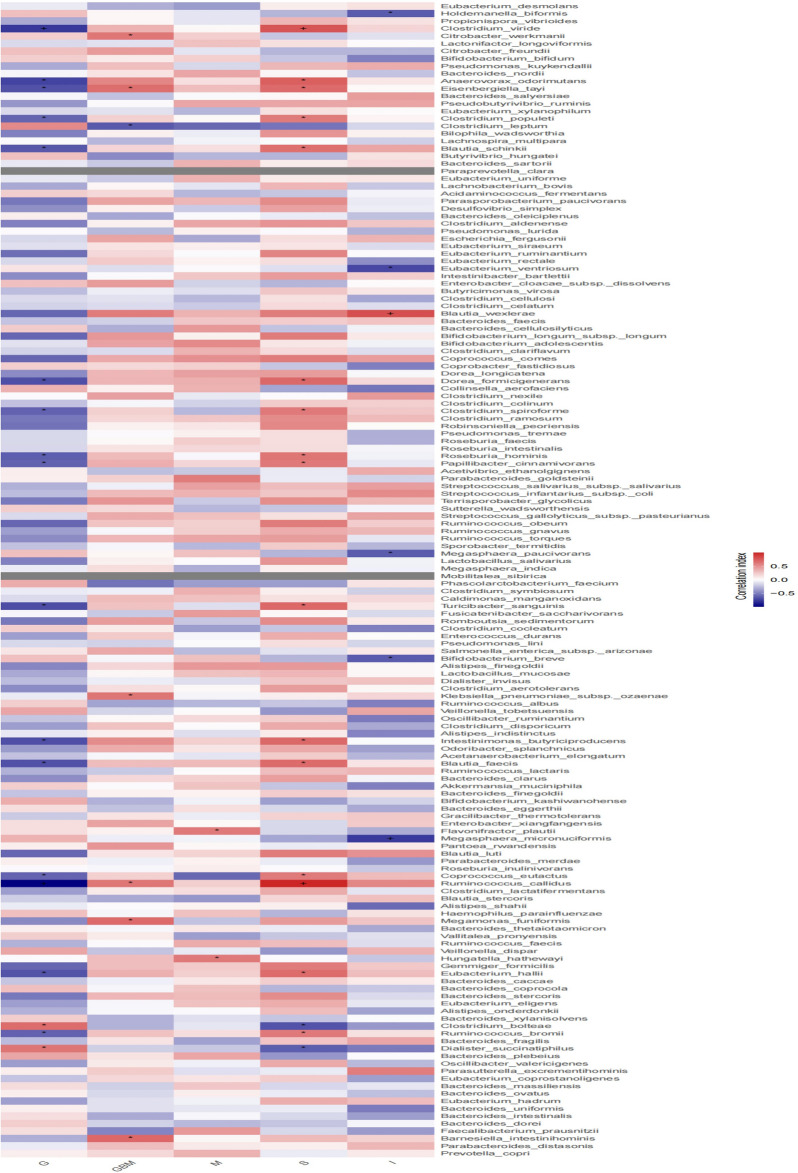
Heatmap showing correlations between genera and 18 pathological classification of diabetic nephropathy. Spearman test, *P < 0.05, ^+^*P* < 0.01.

Correlations between differences in gut microbiota and pathological types were analyzed using spearman correlation analysis. At the genus level, Ruminococcus callidus and Clostridium viride showed strongly negative correlations with glomerular volume and positive correlations with glomerulosclerosis. Thickening of the glomerular basement membrane was positively correlated with Citrobacter werkmanii, Eisenbergiella tay, Klebsiella pneumoniae subsp. ozaenae, Ruminococcus callidus, Megamonas funiformis and Barnesiella intestinihominis. Flavonifractor plautii and Hungatella hatheway were positively correlated with mesangial matrix hyperplasia. Megasphaera micronuciformis showed a positive correlation with renal interstitial lesions (Fig. 5). It is suggested that the changes in the above microbiota lead to glomerular volume reduction, sclerosis, glomerular basement membrane thickening, mesangial matrix hyperplasia and renal interstitial fibrosis, thus promoting the progression of DN. It is suggested that the renal pathological progression of DN may be delayed by regulating changes in the gut microbiota.

## Discussion

Diabetic nephropathy is a common microvascular complication in patients with diabetes. With the sharp increase in the number of diabetic patients^8^, diabetic nephropathy has become the main cause of chronic kidney disease^9^, which constitutes a major public health problem worldwide^10^. A large number of studies have shown that the gut microbiota is closely related to parenteral noncommunicable diseases through gut immunity^11–13^. Qin et al.^14^ and other studies^15^ found a significant decrease in butyrate production in diabetic patients. Wu et al.^16^ found that compared with healthy controls, the relative abundance of Streptococcus and Enterococcus in patients with IgAN was higher, while that of Bacteroides was lower. The changes in the gut microbiota of IgAN affected the metabolism and absorption of polyunsaturated fatty acids and activated the metabolic pathway of arachidonic acid, thus constructing the metabolic system of IgAN. Li et al.^17^ found that the genera Lactobacillus, Clostridium IV, Paraprevotella, Clostridium sensu stricto, Desulfovibrio, and Alloprevotella were enriched in the fecal samples of patients with CKD, while Akkermansia and Parasutterella were enriched in those of NC subjects.

This study found that the α diversity index of patients in the DM and DN groups was higher than that in healthy people, which was consistent with the report of Larsen et al.^18^. As the imbalance pattern of gut microbiota in patients with metabolic diseases is greatly affected by regional factors, there are differences in the imbalance pattern in different regions^19^. Some studies have also found that the α diversity of the gut microbiota in patients with DM decreased^20^. The β diversity analysis showed that there were significant differences among the DM, DN and healthy control groups. Importantly, DM could be accurately distinguished from age- and sex-matched healthy controls by variations in g_Clostridium-XVIII (AUC = 0.929), and DN could be accurately distinguished from age- and sex-matched healthy controls by variations in g_Gemmiger (AUC = 84.2%). DN could be accurately distinguished from age- and sex-matched DM patients by variations in g_Flavonifractor or g_Eisenbergiella (AUC = 0.909 and 0.886, respectively). These results show that the powerful function and practicability of data mining technology are reflected in the diagnosis and prediction of disease markers through a large number of multidimensional genome-related databases. Therefore, future research should continue to look for specific microbial markers for the diagnosis of DN.

Our study found that in healthy people, the content of Clostridium-XVIII was significantly higher than that in the diabetes group. Clostridium-XVIII has the ability to ferment and produce short-chain fatty acids (SCFAs) in the human intestinal tract, and SCFAs are generally considered to play a variety of important roles in maintaining human health. For example, they act as a special nutrition and energy component of the intestinal epithelium, protect the intestinal mucosal barrier, reduce the level of inflammation and enhance gastrointestinal motility^21^. Our findings demonstrated that healthy controls had more Gemmiger in the gut microbiota than DN patients. The correlation analysis between Gemmiger and biochemical indices also found that there was a negative correlation between Gemmiger and blood glucose level. Gemmiger are typically producing butyrate bacteria^22^, which are involved in the adjustment of the body’s reaction to inflammation. This study also found that Eisenbergiella was enriched in gut microorganisms of DN patients, and Eisenberg was positively correlated with urinary microalbumin. The correlation analysis between Eisenbergiella and renal pathological classification of DN showed that Eisenberg was positively correlated with glomerulosclerosis and glomerular basement membrane thickening. This bacterium was highly abundant in the intestinal tract of mice with colitis and might participate in intestinal inflammation^23^. Flavonifractor is a conditional pathogen that was more enriched in DN patients than in the NC and DM groups and synthesizes lipopolysaccharide (LPS). When LPS binds to the receptor complex in macrophages, it activates the signal cascade, resulting in the production of a large number of inflammatory cytokines, such as IL-1β, IL-6, TNF α, and IL-12^24^.

Studies have shown that there is an interaction between intestinal microorganisms and the kidney, called the gut‐kidney axis, and the balance between intestinal symbiotic bacteria and pathogenic bacteria ensures the integrity of the intestinal barrier and normal renal function. Healthy kidneys communicate with the intestinal microbiota through cellular and molecular signals to ensure normal intestinal microbiota homeostasis. The imbalance of DN or gut microbiota will lead to the destruction of this balance, loss of the integrity of the intestinal barrier, activation of immune cells and secretion of cytokines, thus further worsening renal function and symbiotic relationships^25^.

Because diabetic nephropathy is mostly clinically diagnosed, many patients with diabetes complicated with chronic kidney disease are easily misdiagnosed as diabetic nephropathy, resulting in phenotypic heterogeneity. For the accuracy of phenotype, diabetic nephropathy patients confirmed by renal biopsy were selected for the DN group, and eGFR ≥ 60 ml/min/1.73 m^2^ to rule out the effect of end-stage renal disease on gut microbiota^26^. In the DM group, we selected patients with diabetes course of more than 10 years without renal damage, and the difference between the two groups can better reflect the difference of gut microbiota in diabetes and diabetic nephropathy. A number of studies have found that pathological classification of diabetic nephropathy has an important guiding value for judging renal prognosis^27,28^. Therefore, we analyzed the correlation between gut microbiota and pathological indicators of diabetic nephropathy, and found that various gut microbiota play an important role in the pathological progression of diabetic nephropathy. It is suggested that regulating the changes of gut microbiota may delay the progression of renal pathology in diabetic nephropathy.

However, this study is a cross-sectional study of a single-centre and small-scale population, and its results and conclusions have some limitations, so it is necessary to verify them in a follow-up multicentre population cohort. In addition, most of the patients in this study took different types of hypoglycaemic or antihypertensive drugs, so the effect of drugs on microbiota variation also needs to be considered.

Our study explored the imbalance pattern and functional changes of gut microbiota in the two disease states, identified the dangerous strains that promote the progression of diabetic nephropathy in diabetic patients, and established a classification algorithm model to distinguish the two by gut microbiota, providing a certain basis for the diagnosis and prediction of diabetic nephropathy through gut microbiota. The follow-up research can try to restore microbial homeostasis by interfering with gut microbiota, and then protect the kidney by regulating the gut-kidney axis, bringing a new method for the treatment of diabetic nephropathy.

## Data Availability

The datasets analysed during the current study are available in the Sequence Read Archive (SRA) repository, the BioProject ID is PRJNA943281.
